# Thyroid dysfunction in the wake of Omicron: understanding its role in COVID-19 severity and mortality

**DOI:** 10.3389/fendo.2024.1412320

**Published:** 2024-07-16

**Authors:** Qingfeng Zhang, Zongyue Zhang, Xu Liu, Yixuan Wang, Hao Chen, Yueying Hao, Shiqian Zha, Jingyi Zhang, Yang He, Beini Zhou, Ke Hu

**Affiliations:** Department of Respiratory and Critical Care Medicine, Renmin Hospital of Wuhan University, Wuhan, China

**Keywords:** COVID-19, thyroid dysfunction, hypothyroidism, hyperthyroidism, Omicron variant, euthyroid sick syndrome

## Abstract

**Purpose:**

SARS-CoV-2 can invade the thyroid gland. This study was to delineate the risk of thyroid dysfunction amidst the prevalence of the Omicron variant, and to investigate the correlation between thyroid function and Coronavirus disease 2019 (COVID-19) outcomes. The study also aimed to ascertain whether thyroid dysfunction persisted during COVID-19 recovery phase.

**Methods:**

This was a retrospective cohort study. COVID-19 patients from the Renmin Hospital of Wuhan University, China during the epidemic of Omicron variants were included, and their thyroid function were analyzed in groups.

**Results:**

A history of thyroid disease was not associated with COVID-19 outcomes. COVID-19 can lead to a bimodal distribution of thyroid dysfunction. The severity of COVID-19 was inversely proportional to the levels of thyroid- stimulating hormone (TSH), free triiodothyronine (FT3) and free thyroxine (FT4), leading to a higher prevalence of thyroid dysfunction. Severe COVID-19 was a risk factor for euthyroid sick syndrome (ESS) (OR=22.5, 95% CI, 12.1 - 45.6). Neutrophil to lymphocyte ratio mediated the association between severe COVID-19 and ESS (mediation effect ratio = 41.3%, p < 0.001). ESS and decreased indicators of thyroid function were associated with COVID-19 mortality, while high levels of FT3 and FT4 exhibited a protective effect against death. This effect was more significant in women (p < 0.05). During the recovery period, hyperthyroidism was quite uncommon, while a small percentage of individuals (7.7%) continued to exhibit hypothyroidism.

**Conclusion:**

COVID-19 severity was linked to thyroid dysfunction. Severe COVID-19 increased the risk of ESS, which was associated with COVID-19 mortality. Post-recovery, hyperthyroidism was rare, but some individuals continued to have hypothyroidism.

## Introduction

SARS-CoV-2 is the causative agent in COVID-19 (1). The virus’s envelope contains a spike glycoprotein that interacts with the angiotensin-converting enzyme 2 (ACE2) with high specificity and affinity (2). ACE2, a transmembrane protein is found in various organs, including the endocrine system, potentially facilitating the transmission of the virus to these organs. Within the endocrine system, ACE2 is most abundantly present in the testicles, followed by the thyroid and the hypothalamus. The presence of ACE2 in the thyroid renders it a viable target for viral entry (3). Autopsy samples procured from the thyroid gland post-mortem have demonstrated that SARS-CoV-2 can directly infect the thyroid gland, the direct viral insult combined with an intense immune response may trigger or worsen thyroid conditions in predisposed individuals (4, 5).

Thyroid hormones play a pivotal role in regulating the immune system (6). Consequently, COVID-19 could potentially influence thyroid function, and in ture, the state of thyroid function could impact the prognosis of COVID-19.

Existing perspectives suggest that abnormal thyroid function can manifest during the active phase of COVID-19 and persist into the convalescence phase (7). ESS is most prevalent among COVID-19 patients (3) and has been identified as an independent risk factor for disease severity (8). Furthermore, a reduction in FT3 levels has been independently associated with all-cause mortality in patients with severe or critical COVID-19 (9). However, most of the existing research data were collected during the initial phase of the pandemic, when the SARS-CoV-2 variants of concern (VOC) were not yet widespread. Due to the limited sample size, there is a lack of comprehensive studies describing the relationship between COVID-19 and thyroid function. Furthermore, while it is known that both high and low levels of thyroid hormone can be detrimental, no previous studies have explored the non-linear relationship between these hormone levels and the outcomes of COVID-19.

From late 2022 to early 2023, China experienced a pandemic of Omicron variants. Therefore, the aim of this study is to construct a comprehensive map of COVID-19 and thyroid function in a large retrospective cohort during the Omicron variants epidemic.

## Materials and methods

### Subjects and study design

Our study included 1505 patients admitted to Renmin Hospital of Wuhan University in China from December 15, 2022, to January 25, 2023. All individuals had access to serum FT3, FT4, and TSH concentrations, and were diagnosed with COVID-19. Among them, 111 had pre-existing thyroid conditions, while the remaining 1394 did not. COVID-19 outcomes were compared between these two groups. The 1394 patients without thyroid disease were further categorized into four severity groups (mild, moderate, severe, critical) and two outcome groups (survival, death). Thyroid function and diagnostic categories were compared across these groups. For the longitudinal analysis of thyroid function, we examined the patient records in our study for any thyroid function tests conducted prior to their COVID-19 diagnosis (‘Before COVID-19’) and any follow-up thyroid function tests conducted after their initial hospital admission for COVID-19 (‘After COVID-19’). The study was approved by the Ethics Committee of Renmin Hospital of Wuhan University, and informed consent was exempted due to its retrospective nature.

### COVID-19 diagnosis and severity

A COVID-19 diagnosis is confirmed through a real-time reverse transcriptase polymerase chain reaction (RT-PCR) test, using a nasopharyngeal swab. The severity of the disease is classified into four categories: Mild disease: Characterized by mild clinical symptoms without any evidence of pneumonia on imaging. Moderate disease: Defined by the presence of fever and respiratory symptoms, with imaging revealing signs of pneumonia. Severe disease: Diagnosed if any of the following conditions are met: respiratory rate ≥30/min, SpO_2_ ≤ 93% at rest, and >50% progression in 48 hours on imaging. Critical disease: Identified by the occurrence of respiratory failure necessitating mechanical ventilation, shock, or the requirement for admission to an intensive care unit (10).

### Thyroid diagnostic categories

All samples were analyzed using the ADVIA 2400 Automatic Biochemical Analyzer from Siemens, Germany. The reagents for the serum free triiodothyronine (FT3), free thyroxine (FT4), and thyroid-stimulating hormone (TSH) tests were all products of Siemens. Normal ranges are as follows: TSH (0.55-4.78 mIU/L), T3 (2.3-4.2 pg/mL), and T4 (0.89-1.76 ng/dL). Overt hyperthyroidism is defined as a subnormal serum TSH with elevated FT3 and/or FT4. Subclinical hyperthyroidism is defined as a subnormal TSH with normal FT3 and FT4 (11). Overt hypothyroidism is defined as TSH above the reference range and FT3 and/or FT4 below the reference range. Subclinical hypothyroidism is defined by TSH above the reference range and both FT3, FT4 within the normal range (12). Euthyroid sick syndrome (ESS) is characterized by a decreased FT3 and/or FT4 without an increased TSH (8). Euthyroid hyperthyroxinemia/TSH-mediated hyperthyroidism are defined by TSH within or above the reference range and FT3 and/or FT4 above the normal range (11). TSH, FT3, and FT4 are all within the normal range defined as euthyroid.

### Statistical analysis

Continuous variables were expressed as median (IQR) and were compared using the Mann–Whitney U test for two-group comparisons and Kruskal-Wallis test for comparisons among four groups. Categorical variables were presented as absolute values (n) or percentages (%) and were analyzed using the chi-square test. Multivariate logistic regression analyses were conducted to explore the relationships among thyroid disease and outcomes of COVID-19, the severity of COVID-19 and ESS, as well as ESS and mortality. Causal mediation analysis was conducted to examine the relationships between severe/critical COVID-19 and ESS, as well as between severe/critical COVID-19 and mortality. Restricted cubic spline (RCS) analyses were performed to investigate the nonlinear relationships between levels of TSH, FT3, and FT4, and the risk of mortality, separately for males and females. Wilcoxon paired rank sum tests and Friedman test were used for pairwise comparison of TSH, FT3, FT4 in the longitudinal data set. All statistical analyses were performed using R software version 4.3.0 (http://www.r-project.org), and double-sided P < 0.05 was defined as statistical significance.

## Results

### Association between thyroid disease and COVID-19

In the cohort, patients had preexisting thyroid disease had a higher proportion of women compared to the group without thyroid disease (80.2% vs 45.0%). However, no significant disparities were observed in the COVID-19 severity and mortality rates between these two groups (**Table 1**). A multivariate logistic regression analysis revealed that age was risk factor for severe COVID-19 and mortality, while female gender appeared to be a protective factor. A history of thyroid disease, both hyperthyroidism and thyroidectomy/hypothyroidism, did not exhibit any association with the COVID-19 outcomes (**Table 2**).

**Table 1:** Comparative clinical characteristics of groups without and with a history of thyroid disease^*^.

Variables	Without thyroid disease (n = 1394)	Thyroid disease (n = 111)	P value
Age, year	67 (55, 76)	64 (55, 72)	0.308
Female, n (%)	627 (45.0)	89 (80.2)	< 0.001
COVID-19 severity			
*Mild, n (%)*	278 (19.9)	20 (18.0)	0.240
*Moderate, n (%)*	753 (54.0)	70 (63.1)	
*Severe, n (%)*	248 (17.8)	16 (14.4)	
*Critical, n (%)*	115 (8.2)	5 (4.5)	
Death, n (%)	73 (5.2)	3 (2.7)	0.241

*Categorical data shown as number (percentage). Continuous variables displayed as median (interquartile range).

COVID-19, coronavirus disease 2019.

**Table 2:** Multivariate logistic regression analysis for risk of severe/critical COVID-19 and death.

Variables	Severe/critical COVID-19		Death	
	OR (95% CI)	P value	OR (95% CI)	P value
Age	1.06 (1.05 - 1.07)	< 0.001	1.06 (1.04 - 1.08)	< 0.001
Female	0.52 (0.40 - 0.68)	< 0.001	0.55 (0.32 - 0.93)	0.025
Thyroid disease	0.89 (0.50 - 1.50)	0.659	0.68 (0.15 - 2.05)	0.534
*Undergone thyroidectomy or hypothyroidism*	0.82 (0.43 - 1.46)	0.514	0.87 (0.19 - 2.65)	0.830
*Hyperthyroidism*	1.24 (0.35 - 3.82)	0.718	NA	0.982

OR, odds ratio; CI, confidence interval; NA, not available.

### Association between COVID-19 severity and thyroid dysfunction

As COVID-19 severity increased, there were corresponding rises in infection indicators like white blood cell (WBC) count, neutrophil to lymphocyte ratio (NLR), C-reactive protein (CRP), serum amyloid A (SAA), and inflammatory mediators such as interleukin-6 (IL-6) and interleukin-10 (IL-10). Additionally, there was an observed dysfunction in humoral immunity, as indicated by elevated levels of immunoglobulin E (IgE). Conversely, cellular immune function tended to decrease with the severity, as evidenced by lower counts of CD3, CD4, and CD8 T cells. Furthermore, there was a noted decrease in the levels of FT3, FT4, and TSH (**Table 3**).

**Table 3:** Demographic characteristics and laboratory findings across various severity levels and outcomes of COVID-19^*^.

Variables	Total	COVID-19 severity					Survival status		
		Mild (n = 278)	Moderate (n = 753)	Severe (n = 248)	Critical (n = 115)	P value	Survival (n = 1321)	Death (n = 73)	P value
Age, year	1394	53 (37, 65)	66 (56, 74)	74 (66, 82)	78 (68, 84)	< 0.001	66 (54, 75)	79 (68, 84)	< 0.001
Female, n (%)	1394	137 (49.3)	369 (49.0)	79 (31.9)	42 (36.5)	< 0.001	604 (46)	23 (32)	0.017
FT3, pg/mL	1394	3.09 (2.74, 3.51)	2.69 (2.29, 3.09)	2.12 (1.80, 2.46)	1.79 (1.40, 2.08)	< 0.001	2.65 (2.17, 3.11)	1.85 (1.35, 2.14)	< 0.001
FT4, ng/dL	1394	1.21 (1.08, 1.34)	1.18 (1.04, 1.33)	1.19 (1.08, 1.36)	1.07 (0.91, 1.25)	< 0.001	1.19 (1.05, 1.33)	1.10 (0.94, 1.29)	0.006
TSH, uIU/mL	1394	1.636 (1.069, 2.689)	1.588 (0.838, 2.751)	0.876 (0.422, 2.154)	0.696 (0.387, 1.959)	< 0.001	1.472 (0.735, 2.680)	0.696 (0.337, 1.682)	< 0.001
WBC, 10^9^/L	1389	5.52 (4.23, 6.87)	5.60 (4.19, 7.45)	6.89 (4.46, 9.48)	8.81 (5.86, 12.65)	< 0.001	5.76 (4.26, 7.64)	9.14 (5.70, 12.73)	< 0.001
N, 10^9^/L	1386	3.19 (2.40, 4.53)	3.70 (2.54, 5.15)	5.15 (3.23, 7.83)	6.84 (4.36, 11.02)	< 0.001	3.79 (2.61, 5.54)	7.75 (4.25, 11.17)	< 0.001
L, 10^9^/L	1385	1.42 (0.99, 1.86)	1.14 (0.79, 1.55)	0.80 (0.53, 1.18)	0.62 (0.38, 0.89)	< 0.001	1.12 (0.74, 1.56)	0.62 (0.37, 0.83)	< 0.001
NLR	1382	2.33 (1.51, 3.84)	3.16 (2.01, 5.09)	5.94 (3.81, 10.98)	12.78 (6.06, 22.17)	< 0.001	3.36 (2.03, 6)	13.41 (6, 23.92)	< 0.001
CRP, mg/L	968	1.32 (0.50, 8.04)	8.49 (0.71, 31.14)	34.18 (9.37, 75.25)	79.04 (36.22, 120.99)	< 0.001	10.57 (1.07, 42.32)	88.61 (36.28, 141.33)	< 0.001
SAA, mg/L	464	5.60 (5, 27.67)	34.95 (5, 160.78)	152.67 (40.71, 300)	295.42 (100.14, 300)	< 0.001	58.83 (7.31, 248.87)	290.22 (88.66, 300)	< 0.001
ESR, mm/h	111	11 (8, 18)	27 (19, 43)	36 (22, 52)	40 (21, 70)	0.003	27 (18, 48)	36 (18, 66)	0.487
IL-2, pg/mL	222	2.60 (2.35, 2.93)	2.71 (2.43, 3.22)	2.89 (2.30, 3.35)	2.79 (2.42, 3.48)	0.690	2.73 (2.39, 3.22)	2.81 (2.47, 3.62)	0.219
IL-4, pg/mL	222	5.39 (4.71, 5.64)	4.80 (4.38, 5.24)	4.77 (4.35, 5.18)	4.85 (4.53, 5.33)	0.267	4.82 (4.42, 5.27)	4.65 (4.12, 5.29)	0.566
IL-6, pg/mL	224	12.39 (8.21, 25.40)	12.00 (6.07, 35.24)	17.25 (8.40, 54.12)	64.79 (22.15, 186.20)	< 0.001	14.06 (7.31, 45.33)	70.41 (27.15, 269.50)	< 0.001
IL-10, pg/mL	223	5.00 (4.43, 5.17)	5.35 (4.07, 7.45)	5.83 (4.67, 7.91)	8.83 (5.62, 14.21)	< 0.001	5.45 (4.43, 7.66)	9.49 (5.58, 15.89)	0.001
TNF, pg/mL	222	3.45 (2.97, 3.97)	3.60 (2.87, 4.87)	3.30 (2.85, 4.39)	3.12 (2.65, 4.29)	0.488	3.44 (2.84, 4.54)	3.07 (2.63, 4.31)	0.354
IFN, pg/mL	223	2.62 (2.44, 4.47)	2.77 (2.25, 4.04)	2.82 (2.41, 3.97)	2.94 (2.29, 4.58)	0.870	2.76 (2.31, 4.09)	3.19 (2.29, 4.41)	0.386
IL-17, pg/mL	200	2.54 (1.87, 3.69)	2.68 (2.15, 3.89)	2.81 (1.90, 4.66)	2.99 (2.14, 4.70)	0.736	2.68 (2.01, 4.27)	3.37 (2.43, 4.47)	0.262
CD3, n/uL	189	856 (612, 902)	655 (411, 1023)	470 (252, 614)	286 (160, 476)	< 0.001	598 (357, 942)	245 (143, 400)	< 0.001
CD4, n/uL	188	531 (352, 616)	383 (239, 593)	237 (140, 406)	156 (95, 242)	< 0.001	353 (190, 578)	145 (82, 214)	< 0.001
CD8, n/uL	188	276 (196, 413)	234 (142, 389)	156 (93, 304)	102 (65, 155)	< 0.001	202 (132, 353)	89 (62, 148)	< 0.001
CD4/CD8	187	1.53 (1.28, 2.11)	1.60 (1.03, 2.40)	1.61 (1.10, 2.36)	1.64 (0.93, 2.81)	0.986	1.61 (1.03, 2.37)	1.42 (0.93, 2.55)	0.722
CD19, n/uL	188	118 (61, 240)	99 (57, 219)	91 (47, 193)	83 (41, 140)	0.350	97 (56, 215)	68 (41, 137)	0.090
CD16+CD56, n/uL	188	163 (81, 208)	134 (86, 227)	110 (69, 202)	102 (55, 169)	0.065	129 (82, 209)	101 (40, 179)	0.041
IgG, g/L	154	10.19 (7.60, 12.13)	10.40 (8.67, 13.15)	12.05 (9.51, 14.98)	12.10 (9.04, 14.55)	0.126	10.60 (8.67, 13.50)	13.00 (11.85, 14.40)	0.019
IgM, g/L	155	0.95 (0.76, 1.64)	0.95 (0.68, 1.18)	0.64 (0.53, 1.02)	0.96 (0.70, 1.30)	0.035	0.85 (0.63, 1.21)	0.96 (0.71, 1.30)	0.378
IgA, g/L	155	2.12 (1.11, 2.78)	2.15 (1.55, 2.70)	2.92 (1.93, 3.52)	1.69 (1.22, 2.54)	0.023	2.23 (1.55, 2.88)	1.58 (1.18, 2.50)	0.179
IgE, IU/mL	156	25.9 (18.4, 93.7)	40.4 (18.4, 116.0)	130 (29.6, 678.0)	73.3 (22.1, 252.0)	0.012	52.7 (18.4, 176.0)	67.7 (18.4, 246.0)	0.834
C3, g/L	154	0.801 (0.758, 0.967)	0.854 (0.710, 1.040)	0.887 (0.710, 1.123)	0.788 (0.612, 0.897)	0.261	0.852 (0.705, 1.040)	0.780 (0.590, 0.955)	0.169
C4, g/L	153	0.199 (0.174, 0.229)	0.226 (0.184, 0.293)	0.236 (0.181, 0.338)	0.211 (0.159, 0.250)	0.147	0.226 (0,179, 0,293)	0.208 (0.151, 0.235)	0.113

*Categorical data shown as number (percentage). Continuous variables displayed as median (interquartile range).

COVID-19, coronavirus disease 2019; FT3, free triiodothyronine; FT4, free thyroxine; TSH, thyroid-stimulating hormone; WBC, white blood cell; N, neutrophil; L, lymphocyte; NLR, neutrophil to lymphocyte ratio; CRP, C-reactive protein; SAA, serum amyloid A; ESR, erythrocyte sedimentation rate; IL-2, interleukin-2; IL-4, interleukin-4; IL-6, interleukin-6; IL-10, interleukin-10; TNF, tumor necrosis factor; IFN, interferon; IL-17, interleukin-17; CD3, cluster of differentiation 3; CD4, cluster of differentiation 4; CD8, cluster of differentiation 8; CD19, cluster of differentiation 19; CD16, cluster of differentiation 16; CD56, cluster of differentiation 56; IgG, immunoglobulin G; IgM, immunoglobulin M; IgA, immunoglobulin A; IgE, immunoglobulin E; C3, complement component 3; C4, complement component 4.

Out of the total patients, 687 (49.3%) were euthyroid and 430 (30.8%) were diagnosed with ESS. As COVID-19 severity increased, there was a corresponding increase in the proportions of patients with subclinical hyperthyroidism, hypothyroidism and ESS. Specifically, the prevalence of hypothyroidism was 2.5% in mild cases, 5.0% in moderate cases, 6.5% in severe cases, and 8.7% in critical cases (p=0.049). Similarly, the prevalence of ESS was 4.3% in mild cases, 24.4% in moderate cases, 57.3% in severe cases, and 80% in critical cases (p<.001). Interestingly, the proportions of patients with subclinical hypothyroidism decreased with COVID-19 severity. However, the proportions of hyperthyroidism and euthyroid hyperthyroxinemia/TSH-mediated hyperthyroidism were minimal and remained consistent across all severity groups (**Table 4**).

**Table 4:** Thyroid diagnostic categories across various severity levels and outcomes of COVID-19^*^.

Variables	Total (1394)	COVID-19 severity					Survival status		
		Mild (n = 278)	Moderate (n = 753)	Severe (n = 248)	Critical (n = 115)	P value	Survival (n = 1321)	Death (n = 73)	P value
Euthyroid	687 (49.3)	227 (81.7)	408 (54.2)	47 (19.0)	5 (4.3)	< 0.001	681 (51.6)	6 (8.2)	< 0.001
Hyperthyroid	15 (1.1)	2 (0.7)	8 (1.1)	3 (1.2)	2 (1.7)	0.772	14 (1.1)	1 (1.4)	0.556
Subclinical hyperthyroid	101 (7.2)	17 (6.1)	50 (6.6)	30 (12.1)	4 (3.5)	0.007	100 (7.6)	1 (1.4)	0.047
Hypothyroid	71 (5.1)	7 (2.5)	38 (5.0)	16 (6.5)	10 (8.7)	0.049	65 (4.9)	6 (8.2)	0.264
Subclinical hypothyroid	70 (5.0)	9 (3.2)	52 (6.9)	7 (2.8)	2 (1.7)	0.006	69 (5.2)	1 (1.4)	0.175
ESS	430 (30.8)	12 (4.3)	184 (24.4)	142 (57.3)	92 (80)	< 0.001	372 (28.2)	58 (79.5)	< 0.001
Euthyroid hyperthyroxinemia/TSH-mediated hyperthyroidism	20 (1.4)	4 (1.4)	13 (1.7)	3 (1.2)	0 (0)	0.662	20 (1.5)	0 (0)	0.620

*Categorical data shown as number (percentage).

COVID-19, coronavirus disease 2019; ESS, euthyroid sick syndrome; TSH, thyroid-stimulating hormone.

After adjusting for age and sex, the severity of COVID-19 was identified as a risk factor for ESS. Specifically, patients with severe COVID-19 were found to be 22.5 times (95% CI, 12.1 – 45.6) more likely to manifest ESS than those with mild COVID-19 (**Table 5**).

**Table 5:** Multivariate logistic regression analysis for risk of ESS and death.

Variables	OR (95% CI)	P value
Multivariate logistic regression for ESS		
Age	1.01 (1.00 - 1.02)	0.006
Female	0.82 (0.63 - 1.07)	0.143
COVID-19 severity		< 0.001
*Mild*	reference	
*Moderate*	6.16 (3.46 -12.0)	
*Severe*	22.5 (12.1 - 45.6)	
*Critical*	66.8 (32.2 - 150)	
Multivariate logistic regression for Death		
Age	1.04 (1.02 - 1.06)	< 0.001
Female	0.70 (0.41 - 1.18)	0.188
ESS	7.30 (4.10 - 13.8)	< 0.001

OR, odds ratio; CI, confidence interval; COVID-19, coronavirus disease 2019; ESS, euthyroid sick syndrome.

**Figure 1:**
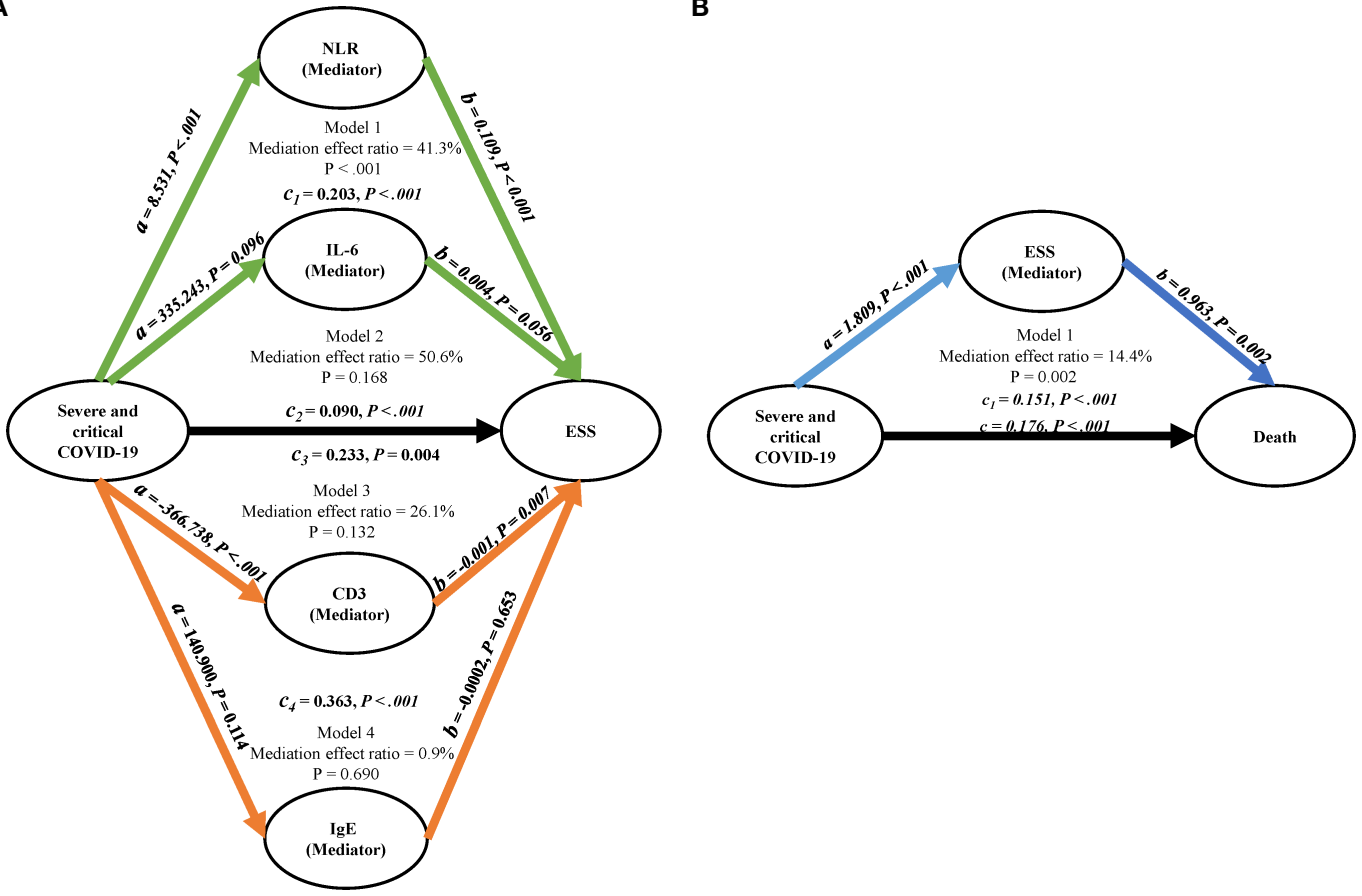
Mediation analysis diagrams. **(A)** Represents the mediation factors NLR, IL-6, CD3, and IgE in the relationship between severe/critical COVID-19 and ESS. **(B)** Illustrates ESS as the mediating factor in the relationship between severe/critical COVID-19 and death; Adjusted for age and gender. COVID-19, coronavirus disease 2019; NLR, neutrophil to lymphocyte ratio; CD3, cluster of differentiation 3; IgE, immunoglobulin E; ESS, euthyroid sick syndrome.

A mediation analysis was performed to explore the potential mediating role of laboratory findings in the relationship between severe/critical COVID-19 and ESS. As depicted in **Figure 1A**, the NLR was found to significantly mediate this relationship, accounting for 41.3% of the effect (p <.001). However, no significant mediating effects were detected for IL-6, CD3, and IgE (all p > 0.05).

### Association between thyroid dysfunction and COVID-19 mortality

As shown in the right section of **Table 3**, the group of COVID-19 patients who did not survive exhibited higher infection indicators and increased levels of inflammatory mediators compared to the group who survived. Furthermore, the non-survival group displayed compromised cellular immune function and diminished thyroid function. However, no significant differences were noted in the humoral immune function between the two groups, except for a mild increase in IgG levels in the non-survival group.

The group of patients who did not survive had a lower proportion of euthyroidism (8.2% vs 51.6%, p<.001) and a higher incidence of ESS (79.5% vs 28.2%, p<.001) compared to the group who survived. Interestingly, the survival group had a higher proportion of subclinical hyperthyroidism (7.6% vs 1.4%, p=0.047). No significant differences were observed in other types of thyroid dysfunction between the two groups (**Table 4**).

After adjusting for age and sex, ESS was identified as a risk factor for COVID-19 mortality (OR = 7.30, 95% CI, 4.10 – 13.8) (**Table 5**). As depicted in **Figure 1B**, ESS was found to significantly mediate the relationship between severe/critical COVID-19 and death, accounting for 14.4% of the effect (p = 0.002).

**Figure 2:**
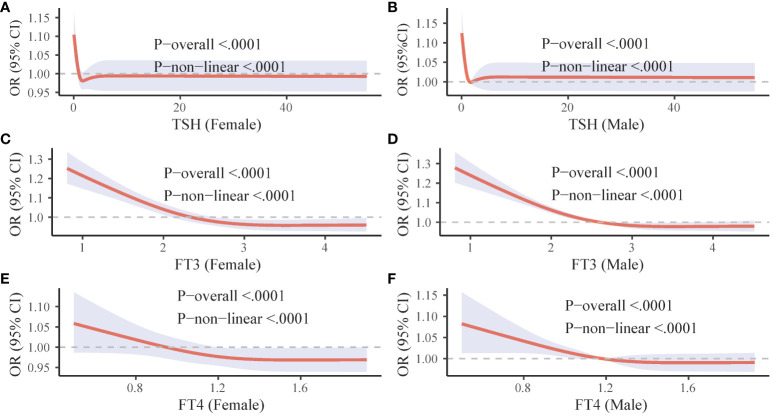
Gender-specific RCS curves for thyroid hormone levels and COVID-19 mortality. The figure is divided into six panels. **(A, C**, and **E** (left column) illustrate the association between TSH, FT3, and FT4 levels respectively, and COVID-19 mortality in females. **(B, D**, and **F** (right column) depict the corresponding associations in males. OR, odds ratio; CI, confidence interval; FT3, free triiodothyronine; FT4, free thyroxine; TSH, thyroid-stimulating hormone.

To investigated the relationship between thyroid function indicators and mortality due to COVID-19, RCS nonlinear correlation curves were constructed separately for male and female populations. These curves reflected the individual correlations between TSH, FT3, FT4, and COVID-19 mortality. In both male and female populations, decreased levels of TSH, FT3, and FT4 (though FT4 was not significant in women) were associated with an increased risk of death from COVID-19. Interestingly, high levels of FT3 were found to have a protective effect against death in both male and female populations, as well as high levels of FT4 in women (**Figure 2**).

### Thyroid function across different time points

**Figure 3:**
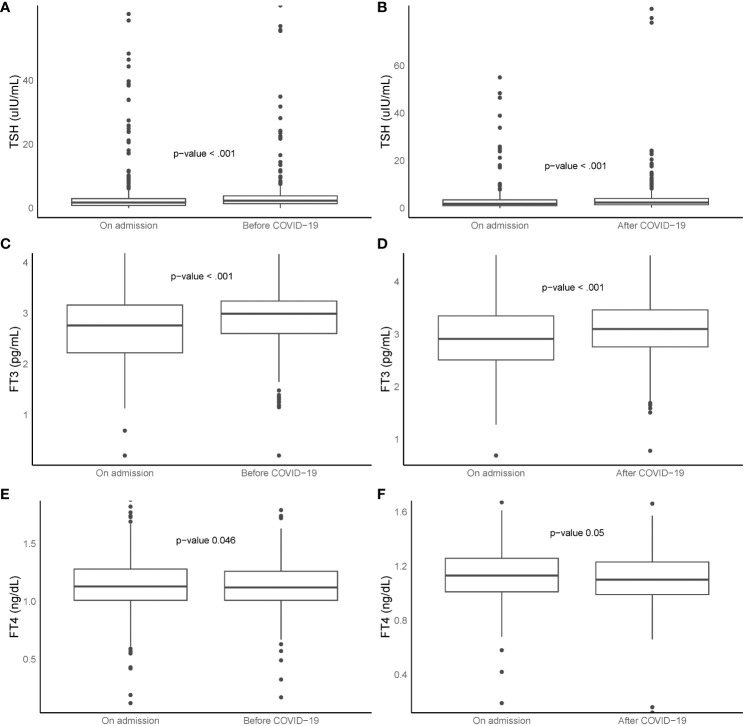
Box plots represent TSH, FT3, and FT4 levels at ‘before COVID-19’, ‘on admission’, and ‘after COVID-19’. Left section **(A, C, E**, n = 411**)**: comparison between ‘before COVID-19’ and ‘on admission’. Right section **(B, D, F**, n = 234**)**: comparison between ‘on admission’ and ‘after COVID-19’. Boxes indicate 25th and 75th percentiles, whiskers indicate 5th and 95th percentiles, and line in box indicates median. Wilcoxon paired rank sum tests used for comparisons. FT3, free triiodothyronine; FT4, free thyroxine; TSH, thyroid-stimulating hormone; COVID-19, coronavirus disease 2019.

Of 1394 patients without prior thyroid disease, thyroid function tests were available for 411 patients before COVID-19 and for 234 survivors during their convalescence. As depicted in the left section (**A**, **C**, **E**) of **Figure 3**, both TSH and FT3 levels at the time of COVID-19 admission were significantly lower than their respective baseline values, while FT4 levels were significantly higher (all p <0.05). Conversely, the right section (**B**, **D**, **F**) of **Figure 3** shows that during the recovery period, both TSH and FT3 levels were significantly higher than those at admission, while FT4 levels were significantly lower (all p <0.05).

During the recovery phase from COVID-19, with a median follow-up of 47 days (ranging from 21 to 98 days), the majority of survivors (70.1%) exhibited euthyroidism. Hyperthyroxinemia and subclinical hyperthyroidism were very rare, with no cases of overt hyperthyroidism observed. However, a small proportion of patients still exhibited ESS (6.8%), hypothyroidism (7.7%), and subclinical hypothyroidism (12.4%) (**Table 6**).

**Table 6:** Thyroid function during COVID-19 convalescence^*^.

Variables	After COVID-19 (n = 234)
Follow-up time, days	47 (21, 98)
FT3, pg/mL	3.10 (2.75, 3.46)
FT4, ng/dL	1.10 (0.99, 1.23)
TSH, uIU/mL	2.360 (1.418, 4.083)
Euthyroid, n (%)	164 (70.1)
Hyperthyroid, n (%)	0 (0)
Subclinical hyperthyroid, n (%)	4 (1.7)
Hypothyroid, n (%)	18 (7.7)
Subclinical hypothyroid, n (%)	29 (12.4)
ESS, n (%)	16 (6.8)
Euthyroid hyperthyroxinemia/TSH-mediated hyperthyroidism, n (%)	3 (1.3)

*Categorical data shown as number (percentage). Continuous variables displayed as median (interquartile range).

COVID-19, coronavirus disease 2019; FT3, free triiodothyronine; FT4, free thyroxine; TSH, thyroid-stimulating hormone; ESS, euthyroid sick syndrome.

**Figure 4:**
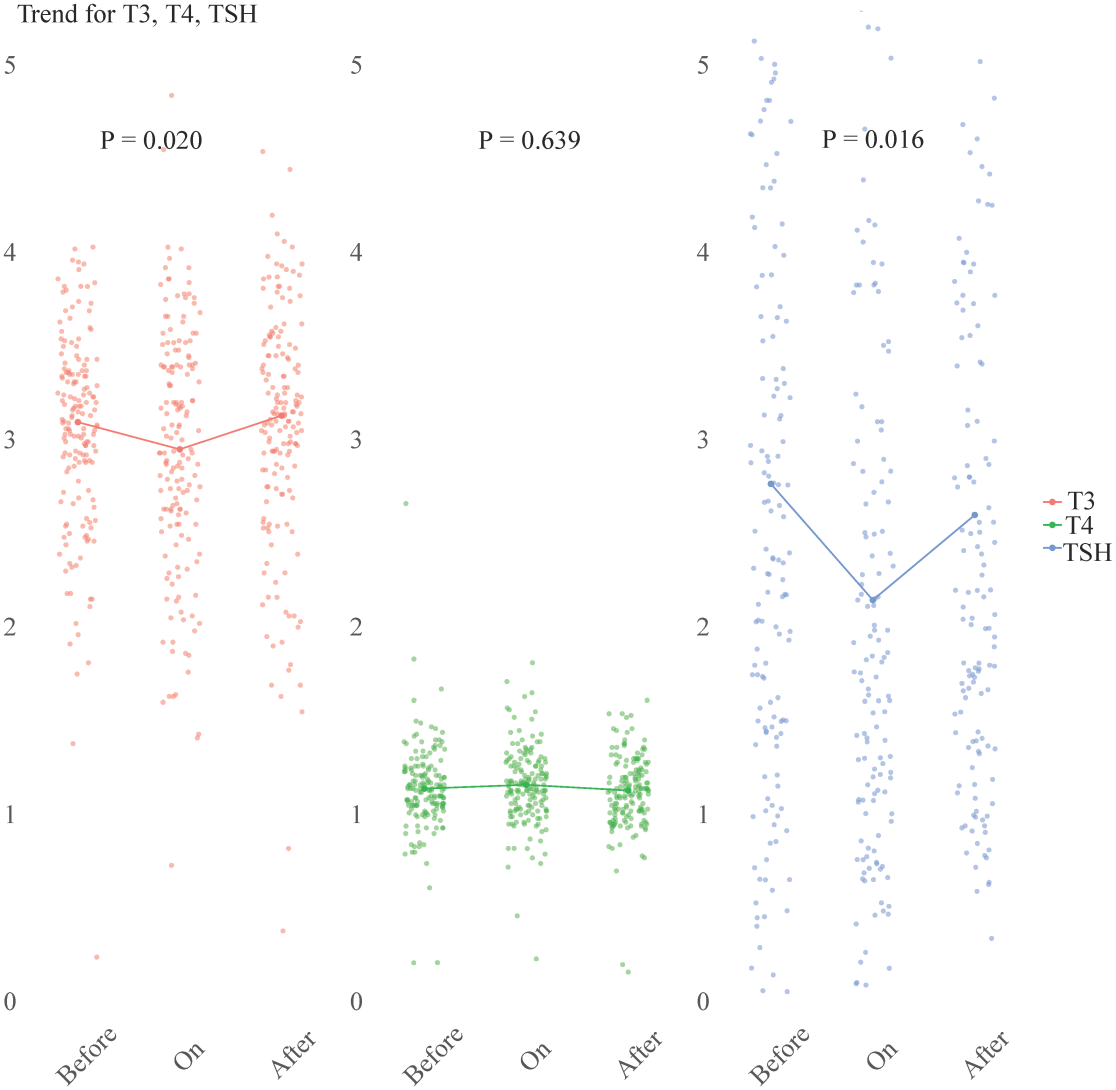
Longitudinal study of TSH, FT3, and FT4 levels at three distinct time points: ‘before COVID-19’, ‘on admission’, and ‘after COVID-19’. The scatter plots (n = 164) represent the individual hormone levels at each time point. Within each section, a line connects the median values of T3, T4, and TSH, providing a visual representation of the central tendency. Statistical analysis was performed using the Friedman test. FT3, free triiodothyronine; FT4, free thyroxine; TSH, thyroid-stimulating hormone.

A subset of 164 patients with complete sets of T3, T4, and TSH measurements taken at three distinct time points: before, during, and after COVID-19. These trends were visually represented in **Figure 4**. The data showed that both FT3 and TSH levels initially decreased upon admission, but eventually returned to their baseline levels during the recovery phase. In contrast, FT4 levels remained relatively stable, with no significant change observed (p = 0.639).

## Discussion

In this study, we found that a prior history of thyroid disease, encompassing both hyperthyroidism and thyroidectomy/hypothyroidism, did not impact the prognosis of COVID-19 during the Omicron variant outbreak. A previous retrospective cohort study involving 3703 COVID-19 patients revealed that 6.8% had pre-existing hypothyroidism. This condition was not associated with an increased risk of hospitalization or mortality (13). But, a recent study has identified the absence of a history of hyperthyroidism as a protective factor against COVID-19 mortality (14). It’s crucial to underscore that in our cohort, among those with a history of thyroid disease, a mere 16 individuals (14.4%) had hyperthyroidism, while the majority 95 individuals (85.6%) had undergone thyroidectomy or were hypothyroidism (not reflected in the tables). Therefore, the conclusions of this study align with those of previous research.

We underscored that COVID-19 severity was inversely related to the plasma levels of TSH, FT3, and FT4, thereby increasing the risk of hypothyroidism and ESS. A meta-analysis encompassing 565 samples from 6 studies revealed that the severe COVID-19 group exhibited significantly lower TSH and FT3 levels compared to the mild group. However, no significant differences were observed in FT4 levels between the groups (15). The variations in FT4 levels observed in our study can be attributed to a more detailed classification of COVID-19 severity. Notably, only the decrease in FT4 levels in critically ill patients reached statistical significance. A recent meta-analysis found that among COVID-19 patients, ESS was the most prevalent thyroid disease with a pooled prevalence of 26%, followed by thyrotoxicosis and hypothyroidism, with pooled prevalences of 10% and 3%, respectively (3). In contrast, our study observed a different pattern. ESS had an incidence of 30.8%, and hypothyroidism was more prevalent than in the meta-analysis. The rate of hyperthyroidism was notably lower at 1.1%. This discrepancy in the prevalence of hyperthyroidism and hypothyroidism may be a characteristic of the Omicron variant infection. Further exploration revealed that COVID-19 severity independently contributes to the risk of ESS. Interestingly, the impact of severe COVID-19 on ESS appeared to be partially mediated through the NLR (mediation effect ratio = 41.3%). Studies have shown that neutrophils exhibited continuous basal hyperactivation in the peripheral circulating blood of COVID-19 patients, which was associated with vascular endothelial injury (16). Neutrophils played a crucial role in the progression of COVID-19 through various mechanisms, including cytokine storms, tissue injury, and thrombotic events (17). In infected thyroid tissues, infiltrates of innate immune cells (macrophages and polymorphonuclear neutrophils) were prevalent (5). In fact, a high NLR was associated with severe COVID-19 and poor prognosis (18). In the context of SARS-CoV-2 infection, it’s currently understood that the immune system may become hyperactive, potentially leading to the development and progression of autoimmune thyroid diseases. This phenomenon might be attributed to abnormal responses of T-cell subtypes, the presence of autoantibodies, and an overproduction of inflammatory cytokines, specifically IL-6, IFN-γ, and TNF-α (19). Our study did observe an increase in the level of inflammatory factors, depletion of cellular immunity, and disorder of humoral immunity with the severity of COVID-19. However, these findings may be limited by the number of patients tested. The roles of IL-6, CD3, and IgE as mediating factors of severe COVID-19 and ESS were not found to be significant.

Although a history of thyroid disease was not found to be associated with COVID-19 outcomes, this study discovered a correlation between thyroid dysfunction resulting from COVID-19 and an increased risk of mortality. Our findings confirmed that ESS was a risk factor for death from COVID-19, and that the high mortality rate of severe COVID-19 could be partially attributed to ESS, with a mediation effect ratio of 14.4%. ESS was typically associated with the severity of the disease and a deteriorating prognosis in critical illnesses. Consistent with previous studies, ESS was associated with severe disease and death from COVID-19, and was considered an early and reliable indicator of poor prognosis for COVID-19 (8, 20). Our study further demonstrated through the RCS curve that across all populations, low TSH and low FT3 increased the risk of death from COVID-19, while high FT3 appeared to have a protective effect. Many previous studies have also observed that low FT3 was more common in patients who died from COVID-19. Low FT3 was associated with excessive inflammation, coagulation, and disorders of the fibrinolytic system, making low FT3 status a risk factor for death (20–23). FT3 was considered to prevent early tissue hypoxia during sepsis, potentially reducing secondary organ failure (24). An ongoing randomized placebo-controlled clinical trial (NCT04348513) aims to investigate whether the administration of T3 (liothyronine, 0.8 g/kg i.v.) to ICU-admitted COVID-19 patients reduces their need for cardiorespiratory support (25). This suggests that our nonlinear model provided a more comprehensive description of the relationship between thyroid hormones and COVID-19 mortality. It’s important to note that the effects of FT3 and FT4 may differ between genders, and the protective effects of FT3 and FT4 may be more pronounced in women. This nuanced understanding of the role of thyroid hormones in COVID-19 outcomes underscores the complexity of this disease and the need for further research.

In paired analysis, we observed that the thyroid function in COVID-19 patients typically decreased upon admission and subsequently returned to baseline during the recovery phase. This observation aligned with previous research, which found that in follow-up studies of COVID-19 survivors, 82.4% (42 out of 51) of abnormal thyroid function tests observed during acute phase of COVID-19 resolved over a span of 6 months (26). Additionally, after a period of 3 or 4 months, COVID-19 patients who underwent pulmonary rehabilitation exhibited an increase in FT3 values (27). Moreover, the thyroid structure also demonstrated spontaneous recovery over time. with 85.7% (6 of 7) patients showing resolved features of thyroiditis after 4 months (28). However, it’s crucial to remain cognizant of the fact that some individuals may continue to experience hypothyroidism.

Our study presents distinct advantages. The substantial sample size of the cohort we included was ample to unveil, for the first time, a comprehensive association between COVID-19 and thyroid function during the Omicron pandemic. However, our research also has its limitations. Firstly, our study lacks data on thyroid autoantibodies and thyroid ultrasound, and dose not address the development of autoimmune conditions post COVID-19. Secondly, this study did not collect information on the underlying disease status and treatment status of COVID-19 patients, with only age and gender employed as confounding factors for analysis and exclusion. It has been demonstrated that obesity is strongly associated with COVID-19 severity and poor outcome, yet our study lacks data on body mass index. Lastly, our study is not prospective and thus cannot establish a definitive causal relationship between COVID-19 and thyroid function.

## Conclusion

Our study delineates a reciprocal relationship between COVID-19 and thyroid function during the Omicron variant pandemic. COVID-19 can detrimentally impact thyroid function, leading to a bimodal distribution of thyroid dysfunction. Inflammation mediates the effects of COVID-19 on ESS, and ESS partially mediates mortality from severe COVID-19. Decreased thyroid function increases the risk of death from COVID-19, while elevated FT3 and FT4 levels appear to confer a protective effect, especially in women. Additionally, there remains a risk of hypothyroidism during the recovery phase from COVID-19. These findings underscore the importance of monitoring thyroid function in COVID-19 patients and highlight the potential therapeutic implications of managing thyroid hormone levels during both treatment and recovery phases.

## Data availability statement

The raw data supporting the conclusions of this article will be made available by the authors, without undue reservation.

## Ethics statement

The studies involving humans were approved by Ethics Committee of Renmin Hospital of Wuhan University. The studies were conducted in accordance with the local legislation and institutional requirements. The ethics committee/institutional review board waived the requirement of written informed consent for participation from the participants or the participants’ legal guardians/next of kin because in accordance with standard research protocols, retrospective studies are typically exempt from the requirement of obtaining written informed consent. This is due to the fact that these studies utilize existing data and do not involve direct interaction with the participants or alterations to their behavior.

## Author contributions

QZ: Writing – original draft, Methodology, Formal analysis, Data curation, Conceptualization. ZZ: Writing – original draft, Methodology, Formal analysis, Data curation, Conceptualization. XL: Writing – review & editing, Resources, Formal analysis. YW: Writing – review & editing, Resources, Formal analysis. HC: Writing – original draft, Formal analysis, Data curation, Conceptualization. YYH: Writing – original draft, Formal analysis, Data curation, Conceptualization. SZ: Writing – original draft, Formal analysis, Data curation, Conceptualization. JZ: Writing – review & editing, Formal analysis. YH: Writing – review & editing, Formal analysis. BZ: Writing – review & editing, Formal analysis. KH: Writing – review & editing, Supervision, Methodology.
